# Associations between Maternal Biomarkers of Phthalate Exposure and Inflammation Using Repeated Measurements across Pregnancy

**DOI:** 10.1371/journal.pone.0135601

**Published:** 2015-08-28

**Authors:** Kelly K. Ferguson, Thomas F. McElrath, Bhramar Mukherjee, Rita Loch-Caruso, John D. Meeker

**Affiliations:** 1 University of Michigan School of Public Health, Department of Environmental Health Sciences, Ann Arbor, Michigan, United States of America; 2 Brigham and Women’s Hospital, Harvard Medical School, Division of Maternal-Fetal Medicine, Boston, Massachusetts, United States of America; 3 University of Michigan School of Public Health, Department of Biostatistics, Ann Arbor, Michigan, United States of America; Institute for Health & the Environment, UNITED STATES

## Abstract

Phthalate exposure is prevalent in populations worldwide, including pregnant women. Maternal urinary metabolite concentrations have been associated with adverse reproductive outcomes, but underlying mechanisms remain unclear. Here we investigate inflammation as a possible pathway by examining phthalates in association with inflammation biomarkers, including C-reactive protein (CRP) and a panel of cytokines (IL-1β, IL-6, IL-10, and TNF-α) in a repeated measures analysis of pregnant women (N = 480). Urinary phthalate metabolites and plasma inflammation biomarkers were measured from samples collected at up to four visits per subject during gestation (median 10, 18, 26, and 35 weeks). Associations were examined using mixed models to account for within-individual correlation of measures. Few statistically significant associations or clear trends were observed, although in full models mono-carboxypropyl phthalate (MCPP) was significantly (percent change with interquartile range increase in exposure [%Δ] = 8.89, 95% confidence interval [CI] = 3.28, 14.8), and mono-benzyl phthalate (MBzP) was suggestively (%Δ = 6.79, 95%CI = -1.21, 15.4) associated with IL-6. Overall these findings show little evidence of an association between phthalate exposure and peripheral inflammation in pregnant women. To investigate inflammation as a mechanism of phthalate effects in humans, biomarkers from target tissues or fluids, though difficult to measure in large-scale studies, may be necessary to detect effects.

## Introduction

Exposure to phthalates diesters occurs ubiquitously in the United States and elsewhere through continual human contact with phthalate-containing materials, particularly flexible plastics, used in vinyl flooring, shower curtains, and food packaging containers, as well as personal care products such as perfumes, deodorants, and nail polish [1]. Phthalates can be absorbed through the skin, lungs, and gastrointestinal tract, and are quickly metabolized into monoesters that are excreted in the urine in unbound or glucuronidated forms [2]. These metabolites are commonly used to assess individual exposures through these various sources and pathways, and have been used to demonstrate widespread exposures in populations worldwide.

Phthalate metabolites are also readily detected in pregnant women [3–5], and the presence of parent compounds and metabolites in umbilical cord blood and amniotic fluid indicate their ability to cross the placental barrier [6, 7]. Exposures to the mother and fetus have been linked to a number of adverse birth outcomes, such as preterm birth and decreased size for gestational age at delivery [8–10], in addition to adverse reproductive [11] and neurodevelopmental outcomes [12] in infants. For a number of these effects, inflammation may be an important underlying mechanism [13]. Some cellular studies have demonstrated that phthalates are capable of inducing pro-inflammatory responses [14, 15], potentially via binding and activation of peroxisome proliferator activated receptors (PPARs) [16]. In humans, several cross sectional studies suggest that phthalate exposure is associated with an increase in biomarkers of systemic inflammation [17, 18].

We investigated whether phthalate exposure during pregnancy was associated with maternal inflammation in a study with repeated measures of exposure and outcome biomarkers. We examined concentrations of urinary phthalate metabolites collected at up to four time points per subject during pregnancy in association with plasma C-reactive protein (CRP), pro-inflammatory cytokines (IL-1β, IL-6, and TNF-α), and an anti-inflammatory cytokine (IL-10). We previously identified associations between urinary phthalate metabolites and the same panel of inflammation biomarkers in a small study of pregnant women in Puerto Rico [19]. The present analysis of a case-control population from Boston expands on our earlier report, and represents the largest study, both in subjects and number of repeated samples, to address this research question in pregnant women to date.

## Materials and Methods

### Study population

Pregnant women included in the present analysis were part of a nested-case control study designed to assess the relationship between maternal phthalate exposure during pregnancy and preterm birth [8]. Subjects for the nested case-control study were selected from a longitudinal cohort of pregnant women (N = 1,181) recruited early in pregnancy who delivered live, singleton infants at Brigham and Women’s Hospital in Boston, MA between 2006 and 2008, and included 132 cases of women who delivered preterm and 350 randomly selected controls. For each subject, urine and plasma samples were collected at up to 4 time points during pregnancy at median 10, 18, 26, and 35 weeks gestation. Participants provided written informed consent at the time of recruitment, and IRB approval for this study was obtained from the University of Michigan and Brigham and Women’s Hospital.

### Measurement of urinary phthalate metabolites

Urine samples were analyzed for a panel of 9 metabolites, including mono (2-ethylhexyl) phthalate (MEHP), mono (2-ethyl-5-hydroxyhexyl) phthalate (MEHHP), mono (2-ethyl-5-oxohexyl) phthalate (MEOHP), mono (2-ethyl-5-carboxypentyl) phthalate (MECPP), mono-benzyl phthalate (MBzp), mono-n-butyl phthalate (MBP), mono-iso-butyl phthalate (MiBP), mono-ethyl phthalate (MEP), and mono (3-carboxypropyl) phthalate (MCPP). Concentrations were measured via mass spectrometry at NSF International (Ann Arbor, MI, USA) using methods adapted from the Centers for Disease Control and Prevention procedures, described in detail by Lewis et al. [20] At the time of phthalate metabolite analysis, urinary specific gravity concentrations were measured using a digital handheld refractometer (Atago Company LTd., Tokyo, Japan) as an index of urine dilution. For statistical analyses, metabolite concentrations below the limits of detection (LOD) were replaced with the LOD divided by the square root of 2.

For examining population distributions, phthalate metabolites were standardized to urinary specific gravity concentrations using the following equation: P_SG_ = P[(1.015-1)/(SG-1)], where P_SG_ represents the specific gravity corrected phthalate concentration, P represents the raw phthalate concentration, 1.015 is the median urinary specific gravity concentration in all samples measured, and SG is the specific gravity of that particular sample [21]. For statistical models, raw phthalate concentrations were examined and specific gravity was included as a covariate. In addition to modeling individual metabolites, we created a molar summed measure of the di (2-ethylhexyl) phthalate (DEHP) metabolites (∑DEHP), including MEHP, MEHHP, MEOHP, and MECPP.

### Measurement of plasma inflammation biomarkers

Plasma CRP was analyzed using a DuoSet enzyme-linked immunosorbent assay (R&D Systems, Minneapolis, MN, USA). Cytokines were assayed using a premixed Milliplex MAP High Sensitivity Human Cytokine Magnetic Bead Panel (EMD Millipore Corp., St. Charles, MO, USA) and measured using a Luminex 200 instrument (Luminex, Austin, TX, USA). All plasma biomarkers were analyzed by the University of Michigan Cancer Center Immunological Monitoring Core (Ann Arbor, MI, USA). Both methods and detection limits have been described in detail elsewhere [22]. As with phthalate metabolites, inflammation biomarker measurements below the LOD were replaced by the LOD divided by the square root of 2 [23].

### Statistical analysis

R version 3.1.0 was used for all statistical analysis [24]. Unless otherwise stated, all analyses were performed with inverse probability weightings to adjust for the case-control study design so that results may be generalized to the parent cohort and to other pregnant populations with similarly distributed demographics [25]. Characteristics of the study participants were examined with percentages by group. Distributions of exposure and outcome biomarkers in the overall sample were examined prior to analysis, with attention to outliers and values below LOD. Skewed distributions of exposure and outcome biomarkers were natural log transformed prior to modeling. To present changes in markers across gestation we calculated medians as well as 25^th^ and 75^th^ percentiles of plasma inflammation biomarkers and specific gravity corrected phthalate metabolites for each study visit. To test for significant differences in biomarker concentrations by study visit we created linear mixed effect models (LME), adjusting for subject-specific random intercepts and slopes, with ln-transformed phthalate or inflammation biomarker concentration predicted by study visit as a factor (to test for significant differences at visits 2, 3, or 4 compared to visit 1) or as a numeric variable (to test for significant linear trends from visits 1–4).

LME were also created to test associations between each inflammation biomarker and each phthalate metabolite measured. Prior to model building, we tested whether to include subject specific random slopes by conducting a likelihood ratio test using restricted maximum likelihood. We also compared Akaike Information Criterion (AIC) values for models with and without inclusion of the random slope. Crude models were adjusted for urinary specific gravity to account for urine dilution. Full models were built by adding covariates one at a time and including in the model if they altered effect estimates by greater than 10 percent. Covariates considered for inclusion as fixed effects were those that were significantly associated with inflammation biomarkers, as previously published [22]. These included maternal race/ethnicity (White, African American, or Other), education level (high school, technical school, junior college or some college, or college graduate/above), health insurance provider (public or private), body mass index (BMI) category at the first study visit (<25 kg/m^2^, 25 to <30 kg/m^2^, or >30 kg/m^2^), and parity (nulliparous or parous). Additionally, we examined time of day of urine sample collection (before vs. after 1pm) as a covariate as this variable can improve the estimation of phthalate concentration. Beta coefficients from crude and full models corresponding to the fixed effects were converted to percent change of inflammation biomarker in association with an interquartile range (IQR) increase in urinary phthalate metabolite for comparison across metabolites and with other studies.

We performed several sensitivity analyses as well. First, we examined full statistical models in cases of preterm birth (i.e., mothers who delivered prior to 37 weeks completed gestation) as well as controls separately (without adjusting for any sampling weight, just as a stratified analysis). We also created models stratified by gender of the fetus, as phthalates have been shown to have gender-specific effects in other studies [26, 27] and placental inflammatory responses may vary based on fetal gender [28]. We also examined associations by study visit, in attempt to identify windows of vulnerability to phthalate exposure. Finally, using generalized additive mixed models (GAMM), we examined potential non-linear associations between longitudinally measured phthalate metabolites and inflammation biomarkers across pregnancy. GAMM were built with the same covariates as LME.

## Results

For the present analysis, subjects were included if they had both urinary exposure and plasma inflammation biomarkers from 1 or more study visits, and time points were excluded if they did not have both measurements. Additionally, one sample was excluded because of an extremely low and influential (changed LME model effect estimates by >10%) CRP concentration for all results presented (final N = 480 subjects, 1521 samples). Weighted maternal demographic characteristics varied only slightly from those previously published on the overall population studied, and are presented in Table 1 [29]. Mothers were median 32.7 years of age (range 18.3–50.2) at their first study visit (visit 1), and over half were white (59%), well-educated (70% with junior college, some college, or a college degree), and of normal or below-normal body mass index at visit 1 (53%). Prevalence of alcohol and tobacco use was also low (5 and 6%, respectively). After weighting, 12% of the sample had a preterm birth, which is roughly equivalent to the rate among the general US population [30].

**Table 1:** Demographic characteristics of weighted study population (N = 480).

Categorical characteristic		Weighted %
Race/ethnicity	White	59
	African American	16
	Other	26
Health Insurance Provider	Private (Private insurance, HMO, self-pay)	81
	Public (Medicaid/SSI/MassHealth)	19
Education	High school	14
	Technical school	16
	Junior college or some college	30
	College graduate	40
Pre-pregnancy BMI	<25 kg/m^2^ (underweight to normal weight)	53
	25-<30 kg/m^2^ (overweight)	27
	≥30 kg/m^2^ (obese to morbidly obese)	20
Tobacco use during pregnancy	Yes	6
	No	94
Alcohol use during pregnancy	Yes	5
	No	95
Parity	Nulliparous	45
	Parous	55
Gender of infant	Male	45
	Female	55
Preterm (delivery <37 weeks gestation)	Yes	12
	No	88

Percentiles of plasma inflammation biomarkers and urinary phthalate metabolite concentrations standardized to urinary specific gravity are shown in Table 2. As previously reported, there were some significant trends in biomarker and phthalate levels across gestation, although levels were relatively stable within individual over time (intraclass correlation coefficients 0.66–0.81) [21, 22]. CRP was slightly higher in plasma samples later in pregnancy, as were IL-6 and TNF-α, and IL-1β decreased slightly between visits 1 and 3 or 4. In regard to urinary phthalate metabolites, ∑DEHP and MCPP decreased across pregnancy, with significantly lower levels at visit 3 compared to visit 1, and levels of MBP and MiBP increased slightly toward the end of gestation [21].

**Table 2:** Median (25^th^, 75^th^ percentiles) concentrations of plasma inflammation biomarkers and urinary phthalate metabolites by study visit in weighted study population.

	Visit 1	Visit 2	Visit 3	Visit 4	p (trend)^a^
N	415	389	363	354	
Gestational age (weeks)	9.57 (8.29, 11.4)	18.0 (17.0, 18.9)	26.1 (25.3, 27.0)	35.1 (34.4, 35.9)	
CRP (μg/mL)	4.14 (2.20, 9.31)	5.88 (3.09, 14.0)*	6.07 (3.26, 13.0)*	5.45 (3.18, 9.42)*	0.002
IL-1β (pg/mL)	0.28 (0.16, 0.52)	0.27 (0.15, 0.47)	0.22 (0.13, 0.50)*	0.24 (0.12, 0.49)*	<0.001
IL-6 (pg/mL)	1.34 (0.80, 2.51)	1.21 (0.74, 2.12)	1.28 (0.80, 2.17)*	1.54 (0.96, 2.52)*	0.195
IL-10 (pg/mL)	13.0 (8.72, 20.2)	13.2 (9.16, 19.4)*	13.4 (9.20, 19.4)	13.5 (9.08, 18.7)	0.401
TNF-α (pg/mL)	2.90 (2.06, 4.07)	2.95 (2.25, 4.28)*	2.98 (2.19, 4.07)*	3.28 (2.24, 4.62)*	<0.001
∑DEHP (μmol/L)	0.38 (0.20, 0.83)	0.32 (0.17, 0.74)*	0.26 (0.13, 0.54)*	0.30 (0.17, 0.94)	0.007
MBzP (μg/L)	6.45 (3.56, 13.8)	6.58 (3.17, 12.8)	5.86 (3.33, 12.1)	7.13 (3.90, 14.8)	0.132
MBP (μg/L)	16.5 (10.9, 26.8)	16.3 (10.3, 28.1)	16.2 (10.4, 26.3)	18.0 (12.4, 30.5)	0.435
MiBP (μg/L)	7.27 (4.65, 11.1)	6.94 (4.52, 11.6)	7.70 (4.62, 11.9)	8.78 (5.66, 14.2)*	0.001
MEP (μg/L)	117 (47.5, 357)	110 (48.5, 375)	130 (50.3, 368)	119 (45.7, 476)	0.995
MCPP (μg/L)	1.72 (1.08, 3.52)	1.74 (1.05, 3.57)	1.55 (0.96, 3.12)*	1.76 (1.06, 3.57)	0.039

*P < 0.05 for difference in biomarker concentration at visit 2,3, or 4 compared to visit 1.

^a^Calculated from linear mixed effect (LME) models with subject specific random intercepts and slopes with biomarker predicted by study visit (continuous)

Results from crude and fully adjusted LME models for the associations between phthalate metabolites and inflammation biomarkers are presented in Table 3. The ∑DEHP associations are presented as results were similar to individual DEHP metabolites (S1 Table). All models were adjusted for random intercepts and slopes, as addition of random slopes significantly improved model fit. Crude models included urinary specific gravity and gestational age at sample collection as covariates. Full models were additionally adjusted for maternal race/ethnicity, health insurance provider, BMI at visit 1, and time of day at urine sample collection. Few statistically significant associations were noted. MCPP was associated with significant higher IL-6 concentrations in both crude (percent change [%Δ] with IQR increase = 8.43, 95% confidence interval [CI] = 3.04, 14.1) and full (%Δ with IQR increase = 8.89, 95% CI = 3.28, 14.8) models. However, a suggestive (p<0.10) inverse association was observed between MCPP and CRP in the full model (%Δ = -4.78, 95% CI = -9.93, 0.67). MBzP was suggestively associated with an increase in CRP (%Δ = 7.62, 95% CI = -0.38, 16.3) and IL-6 (%Δ6.34, 95% CI = -1.25, 14.5) in crude models. In full models the association with IL-6 was still suggestive (%Δ = 6.79, 95% CI = -1.21, 15.4), but for CRP the effect estimate was smaller and less precise (%Δ = 5.71, 95% CI = -2.35, 14.4, p = 0.17). Effect estimates between MiBP and inflammation biomarkers were positive in both crude and full models, but none were statistically significant.

**Table 3:** Percent change (%Δ) and 95 percent confidence intervals (95% CI) in inflammation biomarker in association with an interquartile range increase in urinary phthalate metabolite concentrations during pregnancy.

	Crude Model^a^ (N = 480 subjects, 1518 observations)									
	CRP		IL-1β		IL-6		IL-10		TNF-α	
	%Δ (95% CI)	p	%Δ (95% CI)	p	%Δ (95% CI)	p	%Δ (95% CI)	p	%Δ (95% CI)	p
∑DEHP	-3.09 (-8.23, 2.34)	0.26	2.92 (-2.13, 8.23)	0.26	2.91 (-2.18, 8.25)	0.27	0.94 (-2.76, 4.79)	0.62	-1.65 (-4.20, 0.97)	0.22
MBzP	7.62 (-0.38, 16.3)	0.06	-1.80 (-8.81, 5.76)	0.63	6.34 (-1.25, 14.5)	0.10	2.09 (-3.39, 7.89)	0.46	1.60 (-2.24, 5.60)	0.42
MBP	5.91 (-1.34, 13.7)	0.11	1.05 (-5.56, 8.11)	0.76	2.66 (-4.02, 9.81)	0.44	2.41 (-2.57, 7.65)	0.35	0.80 (-2.66, 4.38)	0.66
MiBP	4.15 (-4.08, 13.1)	0.33	0.61 (-6.95, 8.79)	0.88	4.93 (-2.93, 13.4)	0.23	1.92 (-3.82, 8.01)	0.52	2.41 (-1.67, 6.66)	0.25
MEP	2.03 (-4.63, 9.15)	0.56	-4.44 (-10.4, 1.86)	0.16	4.42 (-2.01, 11.3)	0.18	0.44 (-4.18, 5.29)	0.85	0.65 (-2.63, 4.03)	0.70
MCPP	-3.67 (-8.80, 1.75)	0.18	2.36 (-2.71, 7.7)	0.37	8.43 (3.04, 14.1)	<0.01	2.51 (-1.27, 6.44)	0.20	-0.96 (-3.54, 1.69)	0.48
	Full Model^b^ (N = 464 subjects, 1468 observations)									
	CRP		IL-1β		IL-6		IL-10		TNF-α	
	%Δ (95% CI)	p	%Δ (95% CI)	p	%Δ (95% CI)	p	%Δ (95% CI)	p	%Δ (95% CI)	p
∑DEHP	-2.65 (-7.93, 2.92)	0.34	2.77 (-2.45, 8.27)	0.30	3.00 (-2.29, 8.57)	0.27	1.91 (-1.92, 5.89)	0.33	-1.17 (-3.80, 1.53)	0.39
MBzP	5.71 (-2.35, 14.4)	0.17	0.32 (-7.17, 8.42)	0.94	6.79 (-1.21, 15.4)	0.10	3.25 (-2.49, 9.33)	0.27	1.22 (-2.76, 5.37)	0.55
MBP	4.42 (-2.77, 12.1)	0.24	2.68 (-4.23, 10.1)	0.46	1.92 (-4.94, 9.28)	0.59	2.49 (-2.61, 7.85)	0.35	0.34 (-3.18, 3.99)	0.85
MiBP	2.14 (-6.03, 11.0)	0.62	2.27 (-5.65, 10.9)	0.59	4.00 (-4.06, 12.8)	0.34	1.53 (-4.31, 7.72)	0.62	1.60 (-2.54, 5.92)	0.45
MEP	-0.99 (-7.58, 6.07)	0.78	-3.91 (-10.1, 2.69)	0.24	3.98 (-2.70, 11.1)	0.25	-0.61 (-5.32, 4.34)	0.81	-0.33 (-3.68, 3.13)	0.85
MCPP	-4.78 (-9.93, 0.67)	0.09	2.47 (-2.79, 8.01)	0.36	8.89 (3.28, 14.8)	<0.01	3.44 (-0.47, 7.50)	0.09	-0.69 (-3.34, 2.04)	0.62

Results from weighted linear mixed models with subject-specific random intercepts and slopes.

^a^Crude model adjusted for urinary specific gravity and gestational age at sample collection (N = 480 subjects, 1518 observations);

^b^Full model additionally adjusted for maternal race/ethnicity, health insurance provider, pre-pregnancy body mass index, and time of day of urine sample collection (N = 464 subjects, 1468 observations)

In sensitivity analyses, results stratified by preterm case status, fetal gender, and study visit were largely similar to the results presented in Table 3. However, some deviations were noted. For crude models stratified by case status, inverse associations between ∑DEHP and CRP were statistically significant in cases (%Δ = -12.6, 95% CI = -23.1, -0.75) but were null in controls (%Δ = 1.49, 95% CI = -4.62, 7.98). Also, MiBP was significantly associated with increased CRP in cases (%Δ = 29.4, 95%CI = 7.22, 56.2) but not controls (%Δ = 0.40, 95% CI = -8.62, 10.3). These differences may have been due to instability in case estimates as a result of small sample size (N = 129 subjects, 379 observations). In models stratified by fetal gender, the inverse association between ∑DEHP and CRP was stronger in males (%Δ = -9.05, 95% CI = -17.1, -0.27) compared to females (%Δ = -1.06, 95% CI = -7.98, 6.39) and significant positive associations between MiBP and MEP and CRP were observed in males but not in females.

Finally, when stratified by visit associations between MCPP and IL-6 appeared to be stronger at visit 1 (%Δ = 22.0, 95% CI = 5.01, 41.8) and visit 2 (%Δ = 16.7, 95% CI = -0.55, 36.9) compared to visits 3 (%Δ = 7.05, 95% CI = -9.55, 26.7) and 4 (%Δ = -1.35, 95% CI = -17.6, 18.1).

**Fig 1:**
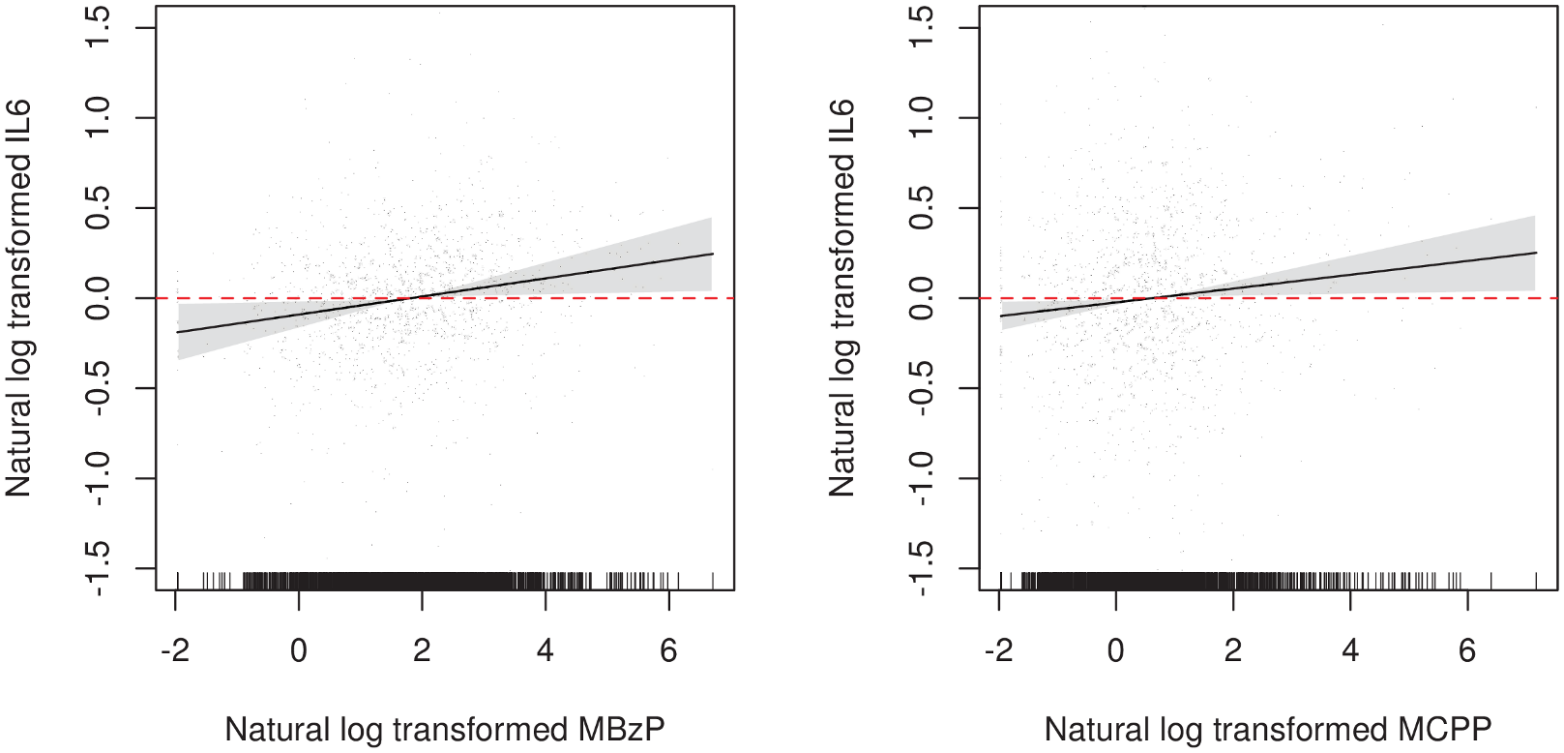
Residual plots from generalized additive mixed models of the associations between mono-benzyl phthalate (MBzP) and mono-carboxypropyl phthalate (MCPP) and IL-6.

We additionally examined the validity of the linearity assumption by using GAMMs. All but one of the associations were linear, as indicated by straight lines and intersecting confidence intervals in GAMM plots of the residuals. As an example, plots of IL-6 with MBzP and MCPP are displayed in Fig 1. The one exception was for the association between MCPP and TNF-α which was primarily flat for lower urinary metabolite concentrations but showed an increasing association at higher concentrations. Nevertheless, confidence intervals included zero at all levels of MCPP (results not shown), suggesting no statistically significant departure from linearity could be established.

## Discussion

In a large study of repeated biomarkers of phthalate exposure and inflammation in pregnant women, we observed few associations. The strongest associations observed were between MCPP levels and increased IL-6, and this association was strongest early compared to late in pregnancy. However, MCPP was not significantly associated with other cytokines indicative of inflammation, and even showed a suggestive inverse association with CRP. Associations between MBzP and MiBP and all inflammation biomarkers were positive, but few reached statistical significance. In general, we found largely null associations between urinary phthalate metabolites and systemic markers of inflammation during gestation.

This evidence is somewhat contrary to previous studies, which suggest that phthalates can cause inflammation in biological systems. Several *in vitro* studies using various cell lines have demonstrated that some phthalates have a pro-inflammatory effect. One study showed that neonatal neutrophils treated with MEHP had increased IL-1β production, and that adult neutrophils treated similarly had increased IL-8 production [15]. In another study, media from cultures of macrophages treated with DEHP had elevated levels of the inflammatory cytokines measured in the present study, including TNF-α, IL-1β, and IL-6, and also increased gene expression for these pathways [31]. However, a third study showed that DEHP treatment of human dendritic cells had anti-inflammatory activity [32]. These studies highlight the fact that inflammatory consequences of phthalate exposure may be highly specific to cell type. While several have hypothesized that inflammatory effects of phthalates may be related to PPAR activation [16], animal studies in this realm focus on the potential adjuvant effect of phthalates. Several studies have demonstrated that rodent exposure to DEHP or MEHP in conjunction with ovalbumin, a protein used to stimulate an allergic reaction, elicits an exacerbated effect compared to exposure to ovalbumin alone [33–36], although there is another report to the contrary [37], and responses and dosages vary by study. Notably, for both *in vitro* and animal studies of phthalate exposure and inflammatory responses, the preponderance of research has explored effects of DEHP or its metabolite MEHP; evidence for consequences of other phthalate exposure is minimal. An adjuvant effect of phthalates may also explain our findings of larger effect estimates for associations between exposure and inflammation biomarkers in mothers who went on to deliver preterm compared to term. Women who deliver prematurely are more likely to have higher levels of other inflammatory stimuli, as this is a well-recognized pathway to preterm birth, and thus phthalate exposure may act to exacerbate the effects of those stimuli.

Cross-sectional human studies have also suggested associations between urinary phthalate metabolites and biomarkers of inflammation. In a large study from the National Health and Nutrition Examination Survey (NHANES), we previously observed significant positive associations between MiBP and MBzP and CRP in a population of male and female children and adults [17]. While in the present study we observed these positive associations as well, they were not statistically significant. Interestingly, in the NHANES study we also observed significant inverse associations between some of the DEHP metabolites and CRP; we observed these associations in the present study as well, though they were not statistically significant. We also previously reported cross-sectional associations with other biomarkers of inflammation in NHANES, including alkaline phosphatase, ferritin, and absolute neutrophil count [18]. The associations were of the highest magnitude between MBP, MiBP, MBzP and MCPP and alkaline phosphatase and absolute neutrophil count, and appeared to have a linear dose-response relationship.

Development of asthma and allergies is the primary endpoint of interest in research devoted to phthalates and inflammation, as apparent from the animal research described above, and by a number of studies in human populations [38]. One repeated measures study examined the association between urinary phthalate metabolites and exhaled nitric oxide, a biomarker of airway inflammation, in 244 inner-city children [39]. The study reported positive associations with MBzP and MEP, but not with MEHHP or MBP when all metabolites were included in the same model. Other epidemiologic studies have suggested associations with asthma and allergic symptoms as well, but many of these have been occupational in nature and focus on descriptive health outcomes rather than biomarkers like those used in our analysis [38].

We previously published findings from a pilot analysis with repeated measures of urinary phthalate metabolites and this same panel of inflammation biomarkers in pregnant women from an ongoing birth cohort in Puerto Rico [19]. That study involved a much smaller sample size (N = 87 subjects, 157 observations from two time points during pregnancy). In that analysis, we observed marginally significant positive associations between MCPP as well as mono (carboxynonyl) phthalate (MCNP; not measured in the present study) and CRP, which is contrary to results from the present study suggesting inverse associations between MCPP and CRP. Also unlike the present study, in the Puerto Rico cohort we observed significant positive associations between DEHP metabolites and MCNP, but not MCPP, and plasma IL-6 concentrations. We also did not see an association between MBzP and IL-6 in that analysis. These inconsistencies may be explained by differences in demographic or other population characteristics across the two cohorts. However, in accordance with the findings from the present study, we observed no statistically significant associations or suggestive patterns between IL-1β or TNF-α and any of the phthalate metabolites measured. In conclusion, the comparison of the associations between these populations highlights MCPP as being the most consistently associated with a systemic inflammatory effect during pregnancy; however the relationships were not reproduced for the same biomarker across the two studies.

There are several explanations for the generally null findings observed in the present study. First, we utilized peripheral (plasma) biomarkers of inflammation. As shown by *in vitro* studies, inflammatory consequences may be very specific to the target cells; effects in the periphery (e.g., expansion of inflammatory cell populations or stimulation of immunoglobulin release) may not be occurring in peripheral cells, and changes in the placenta may not be detectable in maternal circulation. This latter point is supported by research showing that inflammatory cytokines transfer minimally through the fully developed placental barrier [40]. For investigation of birth outcomes, a number of studies have demonstrated that biomarkers in fluids of the gravid reproductive tract (e.g., amniotic or cervicovaginal fluid) are better at predicting adverse pregnancy consequences (e.g., preterm birth) compared with biomarkers in peripheral tissues and fluids [41]. However, it is much more difficult to obtain samples from the reproductive tract during pregnancy, and the present study was not originally designed with these measurements in mind. Alternatively, absence of significant associations may indicate that most phthalates truly do not induce inflammation in pregnant women, at least on the systemic level. It is possible that there is an associated inflammatory response, but it is confined to the intrauterine environment or the maternal-fetal interface. Furthermore, it is important not to generalize these findings to other populations. Pregnant women may have an altered response to inflammatory stimuli; many studies have suggested that pregnancy is a state of immunomodulation in order to avoid rejection of the fetus [42].

Elements of the study design could have contributed to our null findings as well. Such elements include biomarkers selected for assessing inflammation or the timing and stability of exposure and outcome measurements. CRP and the panel of plasma cytokines measured were selected because of their observed associations with preterm birth in previous epidemiologic studies [41], and their utility in examining associations with other environmental chemical exposures such as air pollutants [43]. However, other markers of inflammation more specific to the hypothesized pathways of phthalate induction, such as PPAR gene expression or measuring expansion of specific cell populations, may be a direction for exploration in future research.

Our study had a number of strengths which provide credence to these results. First, we had a large study both in terms of number of subjects and number of repeated measurements of both exposure and outcome biomarkers, which provided ample power for detecting these associations. Second, we used a panel of inflammation biomarkers to encompass multiple potential effects consequences (e.g., inflammatory and anti-inflammatory responses, TH1 and TH2 cytokines).

Third, the assays used to measure markers of both phthalates and inflammation were highly sensitive and thus we had excellent detection rates [21, 22]. Finally, although this analysis was performed on data from a nested case-control study, we applied weightings to statistical models to make the results generalizable to pregnant women in the US.

Of the phthalate metabolites measured in the present study, MCPP had the clearest associations with inflammation biomarkers, and these associations are in concordance with other epidemiologic studies. Animal and cellular studies should be expanded to examine effects of this metabolite and its parent compound, di (*n*-octyl) phthalate, which is still used commonly in plastic products while others like DEHP are being phased out. While we observed few associations overall between other phthalate metabolites and biomarkers of inflammation in peripheral plasma samples, future studies should aim to be more specific to cell type and site. Previous hypotheses argued that maternal inflammation may be a mechanism for the association between phthalate exposure and preterm birth, which we have previously established in this population [8]. However, the present evidence suggests that other pathways may be more relevant for this relationship.

## Supporting Information

S1 Table Percent change (%Δ) and 95 percent confidence intervals (95% CI) in inflammation biomarker in association with an interquartile range increase in urinary di-2-ethylhexyl phthalate metabolite concentrations during pregnancy. (DOCX)

## References

[1] Schettler T. Human exposure to phthalates via consumer products. *International journal of andrology*. 2006, 29, (1), 134–9; discussion 81–5. 16466533 10.1111/j.1365-2605.2005.00567.x

[2] Frederiksen H, Skakkebaek NE, Andersson AM. Metabolism of phthalates in humans. *Molecular nutrition & food research*. 2007, 51, (7), 899–911. 17604388 10.1002/mnfr.200600243

[3] Arbuckle TE, Davis K, Marro L, Fisher M, Legrand M, LeBlanc A, et al Phthalate and bisphenol A exposure among pregnant women in Canada—results from the MIREC study. Environment international. 2014, 68, 55–65. 10.1016/j.envint.2014.02.010 24709781

[4] Tefre de Renzy-Martin K, Frederiksen H, Christensen JS, Boye Kyhl H, Andersson AM, Husby S, et al Current exposure of 200 pregnant Danish women to phthalates, parabens and phenols. *Reproduction*. 2014, 147, (4), 443–53. 24282315 10.1530/REP-13-0461

[5] Zota AR, Calafat AM, Woodruff TJ. Temporal trends in phthalate exposures: findings from the National Health and Nutrition Examination Survey, 2001–2010. *Environmental health perspectives*. 2014, 122, (3), 235–41. 24425099 10.1289/ehp.1306681 PMC3948032

[6] Latini G, De Felice C, Presta G, Del Vecchio A, Paris I, Ruggieri F, et al In utero exposure to di-(2-ethylhexyl)phthalate and duration of human pregnancy. *Environmental health perspectives*. 2003, 111, (14), 1783–5. 14594632 10.1289/ehp.6202 PMC1241724

[7] Silva MJ, Reidy JA, Herbert AR, Preau JL Jr., Needham LL, Calafat AM. Detection of phthalate metabolites in human amniotic fluid. *Bulletin of environmental contamination and toxicology*. 2004, 72, (6), 1226–31. 15362453 10.1007/s00128-004-0374-4

[8] Ferguson KK, McElrath TF, Meeker JD. Environmental phthalate exposure and preterm birth. *JAMA pediatrics*. 2014, 168, (1), 61–7. 24247736 10.1001/jamapediatrics.2013.3699 PMC4005250

[9] Meeker JD, Hu H, Cantonwine DE, Lamadrid-Figueroa H, Calafat AM, Ettinger AS, et al Urinary phthalate metabolites in relation to preterm birth in Mexico city. *Environmental health perspectives*. 2009, 117, (10), 1587–92. 20019910 10.1289/ehp.0800522 PMC2790514

[10] Whyatt RM, Adibi JJ, Calafat AM, Camann DE, Rauh V, Bhat HK, et al Prenatal di(2-ethylhexyl)phthalate exposure and length of gestation among an inner-city cohort. *Pediatrics*. 2009, 124, (6), e1213–20. 19948620 10.1542/peds.2009-0325 PMC3137456

[11] Swan SH, Main KM, Liu F, Stewart SL, Kruse RL, Calafat AM, et al Decrease in anogenital distance among male infants with prenatal phthalate exposure. *Environmental health perspectives*. 2005, 113, (8), 1056–61. 16079079 10.1289/ehp.8100 PMC1280349

[12] Engel SM, Miodovnik A, Canfield RL, Zhu C, Silva MJ, Calafat AM, et al Prenatal phthalate exposure is associated with childhood behavior and executive functioning. *Environmental health perspectives*. 2010, 118, (4), 565–71. 20106747 10.1289/ehp.0901470 PMC2854736

[13] Latini G, Massaro M, De Felice C. Prenatal exposure to phthalates and intrauterine inflammation: a unifying hypothesis. *Toxicological sciences*: *an official journal of the Society of Toxicology*. 2005, 85, (1), 743. 15728702 10.1093/toxsci/kfi131

[14] Jepsen KF, Abildtrup A, Larsen ST. Monophthalates promote IL-6 and IL-8 production in the human epithelial cell line A549. Toxicology in vitro: an international journal published in association with BIBRA. 2004, 18, (3), 265–9. 15046772 10.1016/j.tiv.2003.09.008

[15] Vetrano AM, Laskin DL, Archer F, Syed K, Gray JP, Laskin JD, et al Inflammatory effects of phthalates in neonatal neutrophils. *Pediatric research*. 2010, 68, (2), 134–9. 20453712 10.1203/PDR.0b013e3181e5c1f7 PMC2908957

[16] Hurst CH, Waxman DJ. Activation of PPARalpha and PPARgamma by environmental phthalate monoesters. *Toxicological sciences*: *an official journal of the Society of Toxicology*. 2003, 74, (2), 297–308. 12805656 10.1093/toxsci/kfg145

[17] Ferguson KK, Loch-Caruso R, Meeker JD. Urinary phthalate metabolites in relation to biomarkers of inflammation and oxidative stress: NHANES 1999–2006. *Environmental research*. 2011, 111, (5), 718–26. 21349512 10.1016/j.envres.2011.02.002 PMC3110976

[18] Ferguson KK, Loch-Caruso R, Meeker JD. Exploration of oxidative stress and inflammatory markers in relation to urinary phthalate metabolites: NHANES 1999–2006. Environmental science & technology. 2012, 46, (1), 477–85. 22085025 10.1021/es202340b PMC3258337

[19] Ferguson KK, Cantonwine DE, Rivera-Gonzalez LO, Loch-Caruso R, Mukherjee B, Anzalota Del Toro LV, et al Urinary phthalate metabolite associations with biomarkers of inflammation and oxidative stress across pregnancy in Puerto Rico. *Environmental science & technology*. 2014, 48, (12), 7018–25. 24845688 10.1021/es502076j PMC4066910

[20] Lewis RC, Meeker JD, Peterson KE, Lee JM, Pace GG, Cantoral A, et al Predictors of urinary bisphenol A and phthalate metabolite concentrations in Mexican children. Chemosphere. 2013, 93, (10), 2390–8. 10.1016/j.chemosphere.2013.08.038 24041567 PMC3818401

[21] Ferguson KK, McElrath TF, Ko YA, Mukherjee B, Meeker JD. Variability in urinary phthalate metabolite levels across pregnancy and sensitive windows of exposure for the risk of preterm birth. Environment international. 2014, 70, 118–24. 10.1016/j.envint.2014.05.016 24934852 PMC4104181

[22] Ferguson KK, McElrath TF, Chen YH, Mukherjee B, Meeker JD. Longitudinal profiling of inflammatory cytokines and C-reactive protein during uncomplicated and preterm pregnancy. *American journal of reproductive immunology*. 2014, 72, (3), 326–36. 24807462 10.1111/aji.12265 PMC4573571

[23] Hornung RW, Reed LD. Estimation of average concentration in the presence of nondetectable values. Appl Occup Environ Hyg. 1990, 5, (1), 46–51.

[24] Team RC. R: A Language and Environment for Statistical Computing2014. Available from: http://www.R-project.org.

[25] Jiang Y, Scott AJ, Wild CJ. Secondary analysis of case-control data. Statistics in medicine. 2006, 25, (8), 1323–39. 16220494 10.1002/sim.2283

[26] Kobrosly RW, Evans S, Miodovnik A, Barrett ES, Thurston SW, Calafat AM, et al Prenatal phthalate exposures and neurobehavioral development scores in boys and girls at 6–10 years of age. *Environmental health perspectives*. 2014, 122, (5), 521–8. 24577876 10.1289/ehp.1307063 PMC4014764

[27] Swan SH, Liu F, Hines M, Kruse RL, Wang C, Redmon JB, et al Prenatal phthalate exposure and reduced masculine play in boys. *International journal of andrology*. 2010, 33, (2), 259–69. 19919614 10.1111/j.1365-2605.2009.01019.x PMC2874619

[28] Hodyl NA, Stark MJ, Osei-Kumah A, Clifton VL. Prenatal programming of the innate immune response following in utero exposure to inflammation: a sexually dimorphic process? 2011. 10.1586/eci.11.51 21895471

[29] Ferguson KK, McElrath TF, Chen YH, Mukherjee B, Meeker JD. Urinary Phthalate Metabolites and Biomarkers of Oxidative Stress in Pregnant Women: A Repeated Measures Analysis. *Environmental health perspectives*. 2014. 10.1289/ehp.1307996 PMC4348741 25402001

[30] Martin JA, Hamilton BE, Ventura SJ, Osterman MJ, Mathews TJ. Births: final data for 2011. National vital statistics reports: from the Centers for Disease Control and Prevention, National Center for Health Statistics, National Vital Statistics System. 2013, 62, (1), 1–69, 72. 24974591

[31] Nishioka J, Iwahara C, Kawasaki M, Yoshizaki F, Nakayama H, Takamori K, et al Di-(2-ethylhexyl) phthalate induces production of inflammatory molecules in human macrophages. *Inflammation research*: *official journal of the European Histamine Research Society* [et al]. 2012, 61, (1), 69–78. 10.1007/s00011-011-0390-x 22005928

[32] Kuo CH, Hsieh CC, Kuo HF, Huang MY, Yang SN, Chen LC, et al Phthalates suppress type I interferon in human plasmacytoid dendritic cells via epigenetic regulation. *Allergy*. 2013, 68, (7), 870–9. 23738920 10.1111/all.12162

[33] Guo J, Han B, Qin L, Li B, You H, Yang J, et al Pulmonary toxicity and adjuvant effect of di-(2-exylhexyl) phthalate in ovalbumin-immunized BALB/c mice. PloS one. 2012, 7, (6), e39008 10.1371/journal.pone.0039008 22701742 PMC3373502

[34] Hansen JS, Larsen ST, Poulsen LK, Nielsen GD. Adjuvant effects of inhaled mono-2-ethylhexyl phthalate in BALB/cJ mice. *Toxicology*. 2007, 232, (1–2), 79–88. 17241728 10.1016/j.tox.2006.12.011

[35] He M, Inoue K, Yoshida S, Tanaka M, Takano H, Sun G, et al Effects of airway exposure to di-(2-ethylhexyl) phthalate on allergic rhinitis. *Immunopharmacology and immunotoxicology*. 2013, 35, (3), 390–5. 23672524 10.3109/08923973.2013.787432

[36] Larsen ST, Hansen JS, Hansen EW, Clausen PA, Nielsen GD. Airway inflammation and adjuvant effect after repeated airborne exposures to di-(2-ethylhexyl)phthalate and ovalbumin in BALB/c mice. *Toxicology*. 2007, 235, (1–2), 119–29. 17462807 10.1016/j.tox.2007.03.010

[37] Shin IS, Lee MY, Cho ES, Choi EY, Son HY, Lee KY. Effects of maternal exposure to di(2-ethylhexyl)phthalate (DEHP) during pregnancy on susceptibility to neonatal asthma. *Toxicology and applied pharmacology*. 2014, 274, (3), 402–7. 24370437 10.1016/j.taap.2013.12.009

[38] Kimber I, Dearman RJ. An assessment of the ability of phthalates to influence immune and allergic responses. *Toxicology*. 2010, 271, (3), 73–82. 20371260 10.1016/j.tox.2010.03.020

[39] Just AC, Whyatt RM, Miller RL, Rundle AG, Chen Q, Calafat AM, et al Children's urinary phthalate metabolites and fractional exhaled nitric oxide in an urban cohort. *American journal of respiratory and critical care medicine*. 2012, 186, (9), 830–7. 22923660 10.1164/rccm.201203-0398OC PMC3530221

[40] Aaltonen R, Heikkinen T, Hakala K, Laine K, Alanen A. Transfer of proinflammatory cytokines across term placenta. *Obstetrics and gynecology*. 2005, 106, (4), 802–7. 16199639 10.1097/01.AOG.0000178750.84837.ed

[41] Wei SQ, Fraser W, Luo ZC. Inflammatory cytokines and spontaneous preterm birth in asymptomatic women: a systematic review. Obstetrics and gynecology. 2010, 116, (2 Pt 1), 393–401. 20664401 10.1097/AOG.0b013e3181e6dbc0

[42] Luppi P. How immune mechanisms are affected by pregnancy. *Vaccine*. 2003, 21, (24), 3352–7. 12850338 10.1016/s0264-410x(03)00331-1

[43] Vadillo-Ortega F, Osornio-Vargas A, Buxton MA, Sanchez BN, Rojas-Bracho L, Viveros-Alcaraz M, et al Air pollution, inflammation and preterm birth: a potential mechanistic link. *Medical hypotheses*. 2014, 82, (2), 219–24. 24382337 10.1016/j.mehy.2013.11.042 PMC3928635

