# Correction: Associations between Maternal Biomarkers of Phthalate Exposure and Inflammation Using Repeated Measurements across Pregnancy

**DOI:** 10.1371/journal.pone.0212958

**Published:** 2019-02-22

**Authors:** Kelly K. Ferguson, Thomas F. McElrath, Bhramar Mukherjee, Rita Loch-Caruso, John D. Meeker

In Table 1, the values “Gender of infant” are incorrectly swapped. The first value under “Gender of infant” should be Female and the second value should be Male. Please see the corrected Table 1 here.

**Table 1:** Demographic characteristics of weighted study population (N = 480).

Categorical characteristic		Weighted %
Race/ethnicity	White	59
	African American	16
	Other	26
Health Insurance Provider	Private (Private insurance, HMO, self-pay)	81
	Public (Medicaid/SSI/MassHealth)	19
Education	High school	14
	Technical school	16
	Junior college or some college	30
	College graduate	40
Pre-pregnancy BMI	<25 kg/m^2^ (underweight to normal weight)	53
	25-<30 kg/m^2^ (overweight)	27
	≥30 kg/m^2^ (obese to morbidly obese)	20
Tobacco use during pregnancy	Yes	6
	No	94
Alcohol use during pregnancy	Yes	5
	No	95
Parity	Nulliparous	45
	Parous	55
Gender of infant	Female	45
	Male	55
Preterm (delivery <37 weeks gestation)	Yes	12
	No	88

In the Results, the last sentence of the fourth paragraph is incorrect. The correct sentence is: In models stratified by fetal gender, the inverse association between ∑DEHP and CRP was stronger in females (%Δ = -9.05, 95% CI = -17.1, -0.27) compared to males (%Δ = -1.06, 95% CI = -7.98, 6.39) and significant positive associations between MiBP and MEP and CRP were observed in females but not in males.

## References

[1] Ferguson KK, McElrath TF, Mukherjee B, Loch-Caruso R, Meeker JD (2015) Associations between Maternal Biomarkers of Phthalate Exposure and Inflammation Using Repeated Measurements across Pregnancy. PLoS ONE 10(8): e0135601 10.1371/journal.pone.0135601 26317519 PMC4552851

