# Supplementary material for: Associations between Maternal Biomarkers of Phthalate Exposure and Inflammation Using Repeated Measurements across Pregnancy
Source: PLoS One. 2015 Aug 28;10(8):e0135601. doi: 10.1371/journal.pone.0135601 (PMC4552851; doi:10.1371/journal.pone.0135601)
Supplement: S1 Table — (DOCX) [file pone.0135601.s001.docx]

| **S1 Table.** Percent change (%Δ) and 95 percent confidence intervals (95% CI) in inflammation biomarker in association with an interquartile range increase in urinary di-2-ethylhexyl phthalate metabolite concentrations during pregnancy. | | | | | | | | | | | | | | | | |
| --- | --- | --- | --- | --- | --- | --- | --- | --- | --- | --- | --- | --- | --- | --- | --- | --- |
|  | Crude model^a^ (N=480 subjects, 1518 observations) | | | | | | | | | | | | | | | |
|  | CRP | | | IL-1β | | IL-6 | | | | IL-10 | | | | TNF-α | | |
|  | %Δ (95% CI) | p | | %Δ (95% CI) | p | %Δ (95% CI) | | p | | | %Δ (95% CI) | p | | %Δ (95% CI) | | p |
| MEHP | -0.49 (-5.78, 5.11) | 0.86 | | 3.22 (-1.88, 8.60) | 0.22 | | 2.68 (-2.42, 8.06) | | 0.31 | | 2.90 (-0.91, 6.85) | | 0.14 | | -1.99 (-4.56, 0.64) | 0.14 |
| MEHHP | -1.76 (-6.83, 3.59) | 0.51 | | 2.25 (-2.63, 7.38) | 0.37 | | 2.47 (-2.45, 7.64) | | 0.33 | | 1.12 (-2.49, 4.87) | | 0.55 | | -1.18 (-3.68, 1.38) | 0.36 |
| MEOHP | -1.55 (-6.44, 3.59) | 0.55 | | 2.11 (-2.57, 7.01) | 0.38 | | 2.54 (-2.18, 7.50) | | 0.30 | | 1.36 (-2.11, 4.95) | | 0.45 | | -1.14 (-3.53, 1.32) | 0.36 |
| MECPP | -3.62 (-9.11, 2.19) | 0.22 | | 3.71 (-1.78, 9.51) | 0.19 | | 3.54 (-1.96, 9.36) | | 0.21 | | 0.19 (-3.78, 4.32) | | 0.93 | | -1.86 (-4.61, 0.96) | 0.19 |
|  | Full model^b^ (N=464 subjects, 1468 observations) | | | | | | | | | | | | | | | |
|  | CRP | | | IL-1β | | IL-6 | | | | IL-10 | | | | TNF-α | | |
|  | %Δ (95% CI) | | p | %Δ (95% CI) | p | %Δ (95% CI) | | p | | %Δ (95% CI) | | p | | %Δ (95% CI) | | p |
| MEHP | 0.34 (-5.13, 6.13) | | 0.91 | 3.30 (-2.02, 8.91) | 0.23 | 2.51 (-2.82, 8.13) | | 0.36 | | 3.73 (-0.22, 7.83) | | 0.07 | | -1.59 (-4.25, 1.13) | | 0.25 |
| MEHHP | -1.12 (-6.36, 4.41) | | 0.68 | 2.24 (-2.84, 7.57) | 0.40 | 2.46 (-2.68, 7.87) | | 0.36 | | 2.07 (-1.67, 5.96) | | 0.28 | | -0.69 (-3.28, 1.96) | | 0.61 |
| MEOHP | -1.10 (-6.14, 4.21) | | 0.68 | 2.07 (-2.79, 7.17) | 0.41 | 2.63 (-2.32, 7.82) | | 0.30 | | 2.25 (-1.35, 5.98) | | 0.22 | | -0.72 (-3.20, 1.82) | | 0.57 |
| MECPP | -3.36 (-8.93, 2.56) | | 0.26 | 3.44 (-2.20, 9.41) | 0.24 | 3.80 (-1.92, 9.85) | | 0.20 | | 1.12 (-2.97, 5.37) | | 0.60 | | -1.49 (-4.30, 1.41) | | 0.31 |

Results from weighted linear mixed models with subject-specific random intercepts and slopes. ^a^Crude model adjusted for urinary specific gravity and gestational age at sample collection (N=480 subjects, 1518 observations). ^b^Full model additionally adjusted for maternal race/ethnicity, health insurance provider, pre-pregnancy body mass index, and time of day of urine sample collection (N=464 subjects, 1468 observations).
